# Plant synthetic biology: from knowledge to biomolecules

**DOI:** 10.3389/fpls.2025.1562216

**Published:** 2025-10-28

**Authors:** Soyoung Park, Vimalraj Mani, Kihun Ha, Jin A. Kim, Sichul Lee

**Affiliations:** Department of Agricultural Biology, National Institute of Agricultural Sciences, Rural Development Administration, Jeonju, Republic of Korea

**Keywords:** plant synthetic biology, functional biomolecules, CRISPR/Cas, metabolic engineering, large-scale production

## Abstract

Plant synthetic biology is rapidly emerging as an innovative approach to solving complex problems in human health and agriculture. Although conventional metabolic engineering primarily focuses on microbial systems for large-scale biomolecules production, these platforms often face limitations in expressing plant-derived enzymes and synthesizing structurally complex molecules. In contrast, recent advances in plant synthetic biology have integrated multidisciplinary tools, from molecular biology and biochemistry to synthetic circuit design and computational modeling, to engineer plant systems with enhanced traits. These include improved yield, nutritional quality, environmental resilience, and synthesis of pharmaceutically relevant functional biomolecules. This review focuses on the fundamental technologies that have enabled such advances, which include DNA synthesis, programmable gene circuits, and CRISPR/Cas-based genome editing. We discussed recent applications of reprogramming plant metabolic pathways and existing obstacles, such as transformation efficiency, regulatory bottlenecks, and pathway instability. This review provides key case studies and a forward-looking perspective on the evolution of plant synthetic biology as a robust foundation for sustainable biomanufacturing and production of functional biomolecules.

## Introduction

In recent decades, synthetic biology has emerged as a transformative approach for engineering biological systems to address the global challenges in agriculture, medicine, and bioenergy (Rizzo et al., 2023). Initially, the field focused on using genetically tractable microbial platforms such as *Escherichia coli* and *Saccharomyces cerevisiae* to produce valuable plant natural products (PNPs) (Martin et al., 2003). These microbial systems enable the efficient screening of enzyme combinations and pathway reconstruction. For example, terpenoid precursors have been successfully produced in *E. coli* by engineering the mevalonate pathway (Martin et al., 2003), which has enabled scalable biosynthesis of plant natural products through synthetic regulatory circuits and dynamic control elements. Despite their utility, microbial systems in synthetic biology face challenges such as the toxicity of target compounds, which can impair cell growth, stability, and yield (Han and Miao, 2024), as well as suboptimal metabolic flux and inherent limitations in biosynthetic capacity (Kim et al., 2025). Moreover, microbial systems, particularly prokaryotes, face significant limitations in synthetic biology due to their inability to perform complex eukaryotic post-translational modifications, which hinders the production of properly modified proteins (Brunk et al., 2018). Challenges also arise in producing plant-derived metabolites because of metabolic burden and the absence of necessary biosynthetic pathways (Wu et al., 2016).

Plant-based chassis is gaining recognition as a vital platform in synthetic biology because it naturally accommodates intricate metabolic networks, compartmentalized enzymatic processes, and unique plant biochemical environments that are challenging to replicate entirely in microbial systems (Han and Miao, 2024). This integration facilitates the production of structurally complex metabolites that are otherwise difficult to replicate (Lin et al., 2023; Gharat et al., 2025; Grzech et al., 2023; Zhu et al., 2021).

Plant synthetic biology, defined as the application of engineering principles to plant systems, has seen significant growth since the mid-2000s (Andrianantoandro et al., 2006). Plant synthetic biology necessitates the integration of advanced technologies such as synthetic circuit design, genome editing, and omics-driven pathway discovery, while also considering unique plant-specific features including vacuolar sequestration, tissue-specific gene expression, and plastidial compartmentalization. These features are critical for the biosynthesis of structurally complex metabolites (Han and Miao, 2024; Jores et al., 2021). Recent technological advancements, including cost-effective DNA synthesis and precise genome editing systems, have greatly improved the feasibility of reprogramming plant hosts to enhance or introduce novel biosynthetic capabilities, thereby enabling scalable production of pharmaceutically relevant compounds directly within plant chassis (Baltes and Voytas, 2015; Sadre, 2024; Yang et al., 2022).

**Figure 1:**
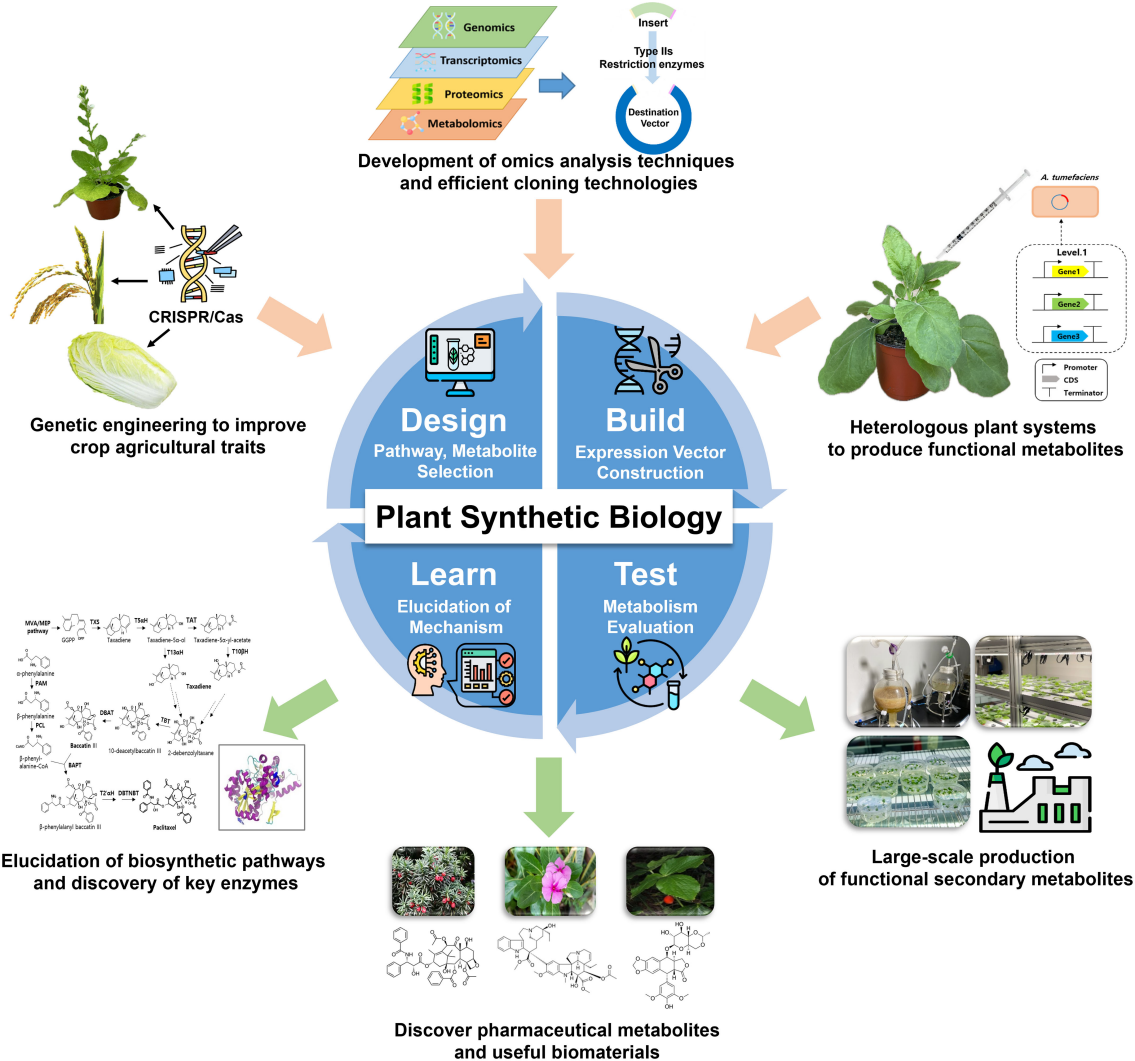
Schematic representation of the core technologies and their applications in the field of plant synthetic biology. Multi-omics data guides the design of biosynthetic pathways from crops and medicinal plant sources. In the Build phase, expression vectors are assembled and introduced into chassis like *Nicotiana benthamiana* via *Agrobacterium*. The Test phase evaluates metabolite yield and stability using LC-MS or GC-MS in tissue culture or greenhouse systems. The Learn phase applies computational tools to refine pathway design and overcome regulatory bottlenecks, aiming for scalable production of functional biomolecules such as flavonoids and alkaloids.

Applications of plant synthetic biology range from improving yield, stress resistance, and nutritional content to enabling *de novo* biosynthesis of anticancer, anti-inflammatory, and neuroactive agents directly in plant or plant-compatible chassis systems (Zhang et al., 2025). Compared to traditional breeding or microbial metabolic engineering, these plant-based strategies provide biologically compatible platforms that overcome issues related to enzyme expression, toxicity, and structural complexity, thus offering promising alternatives for the sustainable bioproduction of therapeutics and nutraceuticals (Han and Miao, 2024). Instead of concentrating exclusively on modifications at the level of individual genes, contemporary strategies prioritize the reconfiguration of metabolic systems. This is achieved through Design-Build-Test-Learn (DBTL) frameworks, which facilitate predictive modeling and systematic enhancement of biosynthetic capabilities (Goshisht, 2024; Lin et al., 2023) (**Figure 1**).

This review highlights key technological advancements in plant synthetic biology, primarily from 2020 to 2024, while incorporating insights from studies conducted in early 2025, with a focus on genome editing, combinatorial pathway engineering, and functional genomics. Emphasis is placed on recent case studies that demonstrate how plant systems are being developed into programmable bio-factories for efficient and scalable production of pharmaceutically relevant functional biomolecules. By bridging foundational concepts and application-focused strategies, this review aimed to clarify the distinct advantages of plant-based systems and inspire future innovations in therapeutic biosynthesis.

## Integrating omics and genome editing for functional pathway engineering

The integration of omics technologies with genome editing tools has opened a new era in metabolic pathway engineering, enabling the precise and efficient production of valuable natural compounds in plants and microbes (Kumar et al., 2021). Combining the comprehensive, systems-level insights provided by omics with the targeted manipulation capabilities of CRISPR/Cas-based genome editing enables researchers to identify, modify and optimize complex biosynthetic pathways, enhancing existing metabolites or generating novel compounds (Hassan et al., 2021; Kumar et al., 2021; Singh et al., 2022).

Omics technologies including genomics, transcriptomics, proteomics, and metabolomics, offer comprehensive data on gene expression, protein function, and metabolite profiles (Dai and Shen, 2022). These data-driven platforms enable the reconstruction of entire biosynthetic networks and the identification of key regulatory points. For example, metabolomics can reveal the accumulation patterns of secondary metabolites, while transcriptomics helps identify gene clusters responsible for their biosynthesis (Wang et al., 2025).

Once candidate genes or regulatory elements have been identified, genome editing tools such as CRISPR/Cas9, base editors or prime editors can be used to knock out, activate or fine-tune the target genes (Ma et al., 2023). This integrative approach allows biosynthetic pathways to be engineered in a rational way, tailored to specific production goals.

Glutamate decarboxylase (GAD), a key enzyme in GABA biosynthesis, exists in five genes in tomatoes. Among these, two (*SlGAD2* and *SlGAD3*) are expressed during fruit development. To increase GABA content in tomatoes, CRISPR/Cas9 technology was used to edit these two genes (Nonaka et al., 2017). As a result, GABA accumulation increased by 7- to 15-fold, indicating that targeted genome editing can enhance the accumulation of functional compounds. Chavez et al. (2022) conducted a co-expression analysis of transcriptomic and metabolomic data to identify candidate genes involved in tropane alkaloid biosynthesis, followed by functional validation of these candidate genes in yeast. This integrated approach significantly accelerated pathway discovery by efficiently decoding complex plant pathways and overcoming the traditional bottleneck of labor-intensive genetic mutant screening. Although omics-integrated discovery is powerful, downstream in planta validation remains essential for pathway fidelity.

Plant transformation remains a critical prerequisite for the effective application of genome editing technologies. However, its implementation is often hindered by gene delivery and regeneration barriers, which vary considerably across species and genotypes (Altpeter et al., 2016). Future research is, therefore, expected to focus on overcoming these limitations through advances in tissue culture methodologies, the development of novel transformation strategies, and the establishment of genotype-independent delivery systems (Altpeter et al., 2016; Ma et al., 2023). Together, these integrative omics and genome-editing strategies not only identify candidate genes with high confidence but also establish the foundation for their functional assembly into synthetic pathways. The following section explores how these discoveries transition into pathway reconstruction for compound production.

## Identification and reconstruction of the biosynthetic pathways for natural products derived from plants

Advancements in omics technologies have significantly accelerated our understanding of plant metabolic networks. Owing to their structural diversity and bioactivity, plant secondary metabolites (PSMs) are critical components in pharmaceutical, nutraceutical, and cosmetic industries (Yeshi et al., 2022). Bioinformatics methods and systems biology approaches have proven effective in identifying correlations between metabolite production and gene expression related to PSM biosynthesis. These tools are particularly valuable for understanding the complex biological relationships in plants (Al Aboud, 2024). Integrated omics and bioinformatics pipelines are now routinely used to map these responses to gene function, enabling pathway mining even in non-model species (Shen et al., 2023; Singh et al., 2022).

Heterologous systems are essential for reconstructing and validating the biosynthetic pathways of natural products identified through omics research (Luo et al., 2015). These systems enable researchers to express biosynthetic gene clusters from various organisms in genetically tractable hosts, facilitating functional validation of gene activity and production of target metabolites that are often undetectable in the original source organism. Among plant-based hosts, *Nicotiana benthamiana* has become a popular platform for several reasons, including its large leaves, rapid biomass accumulation, simple and efficient *Agrobacterium*-mediated transformation, high transgene expression levels, and the availability of extensive literature and standardized protocols (Golubova et al., 2024; Jiang et al., 2024; Li et al., 2019).

Transient expression systems using *N. benthamiana* have enabled the rapid reconstruction of biosynthetic pathways for a wide range of valuable plant-derived compounds, including flavonoids such as diosmin (Lee et al., 2024a) and chrysoeriol (Lee et al., 2024b), costunolide and linalool (Park et al., 2022), triterpenoid saponins (Reed et al., 2023), and anticancer precursors such as paclitaxel intermediates (Zhang et al., 2023). For example, diosmin biosynthesis requires the coordinated expression of five to six flavonoid pathway enzymes, including dioxygenases and methyltransferases, producing diosmin at up to 37.7 µg/g fresh weight (FW) in *N. benthamiana* leaves under transient expression conditions (Lee et al., 2024a). Reed et al. (2023) exemplified this by reconstructing the biosynthesis of QS-7 saponin, a class of vaccine adjuvants, via the co-expression of 19 pathway genes, including multiple cytochrome P450s and glycosyltransferases, achieving 7.9 µg/g dry weight (DW) yields in planta. These cases demonstrate the potential of plant chassis to outperform microbial systems by providing a compatible cellular environment for complex post-translational modifications and metabolite compartmentalization, processes often limited in microbial hosts, thereby enabling the biosynthesis of structurally complex metabolites. To realize this potential at an industrial scale, priority should be given to improving infiltration efficiency, optimizing vector design, enhancing host traits, and integrating stable chassis lines to ensure continuous yields.

Despite its versatility as a heterologous and transient expression platform in synthetic biology, *N. benthamiana* faces several challenges. These include complex metabolic networks that cause side reactions, immune responses, transcript silencing, endoplasmic reticulum (ER) stress and unwanted modifications, reducing yield and complicating purification processes (Alvisi et al., 2021; Beritza et al., 2024; Sukenik et al., 2018). However, these challenges can be overcome by reducing the metabolic background, using inducible or tissue-specific expression, suppressing immune responses and gene silencing, performing glycoengineering and precise genome editing, and integrating high-throughput screening with automated cultivation. Together, these approaches can enhance the platform’s potential for sustainable natural product biosynthesis.

In synthetic biology, a variety of alternative platforms beyond *N. benthamiana* such as microbial hosts, other plant species, moss, algae, and cell-free systems, are actively employed to complement existing methods. For example, the functional roles of key artemisinin biosynthesis genes such as *AaADS* and *CYP71AV1* have been successfully reconstituted in heterologous host plants (Firsov et al., 2021). Additionally, *Salvia miltiorrhiza*, the use of an artificial transcription factor (*SmMYB36*-VP16) in a hairy root system significantly enhanced the yields of tanshinones and phenolic acids (Jia et al., 2024).

## Large-scale production of advantageous metabolites through the strategic modification of metabolic pathways

Strategic modification of metabolic pathways by synthetic biology enables the scalable and sustainable production of valuable metabolites, which has significant applications in pharmaceuticals, biofuels, nutraceuticals, and specialty chemicals (Guo et al., 2017). PSMs such as complex alkaloids, terpenoids, and flavonoid glycosides, are known for their notable pharmaceutical properties. Recent developments in plant synthetic biology have made it possible to reconstruct biosynthetic pathways in heterologous plant hosts, offering new opportunities for the sustainable production of essential pharmaceuticals as recognized by the World Health Organization (WHO) (Sadre, 2024). Although chemical synthesis is feasible, it is resource-intensive and limited by the structural complexity of many PSMs. This has led to increased interest in synthetic biology as a viable alternative for metabolite production (Han and Miao, 2024).

Artemisinin production in *Artemisia annua* has been successfully increased using synthetic biology, which reprograms the plant’s native biosynthesis route (Hassani et al., 2023). Through *Agrobacterium*-mediated transformation in *A. annua*, important enzymes and trichome-specific transcription factors were co-overexpressed, increasing artemisinin content in T_0_ lines by up to 3.2-fold and in T_1_ progeny by roughly 2.2-fold. These results suggested that transcription factor–guided metabolic engineering can enhance medicinal plant yields, offering a stable, cost-effective artemisinin supply. Further improvements may come from targeting pathway bottlenecks and trichome development.

Heterologous expression platforms, such as *N. benthamiana*, which allow for rapid and high-yield transient expression, have been widely adopted to produce specialized metabolites (Molina-Hidalgo et al., 2021). Coupled with *Agrobacterium*-mediated infiltration, these systems enabled the delivery of biosynthetic gene cassettes and combinatorial pathway testing. These strategies can be broadly categorized into transcriptional regulation and modular pathway reconstruction. In transcriptional regulation strategies, transient expression of *VmMYBA1* and *VmMYBA2* from bilberry in *N. benthamiana* leaves induced anthocyanin accumulation like delphinidin-3-rutinoside and activated expression of dihydroflavonol 4-reductase (*DFR*) and anthocyanidin synthase (*ANS*) genes for flavonoid biosynthesis (Karppinen et al., 2021).

On the other hand, modular reconstruction strategies, such as the synthesis of glucoraphanin (Barnum et al., 2022), monoterpenoid indole alkaloid (MIA) precursors (Dudley et al., 2022), and complex pathway stacking (Reed et al., 2023; Jia et al., 2024), have broadened the diversity and scale of metabolites that can be produced in plants.

In addition, cannabinoids and their glucosides are synthesized in *N. benthamiana* and yeast through the transient expression of multi-gene biosynthetic pathways (Gülck et al., 2020). These studies underscore the complementary strengths of plant and microbial platforms for pathway reconstruction and reveal important issues such as metabolite instability, off-target glycosylation, and scalability, which differ based on the compound class and host plant context (Gülck et al., 2020; Lee et al., 2024a).

Despite these encouraging experimental findings, translation to industry remains limited. The flexibility and scalability of plant-based systems have been demonstrated to produce complex bioactive molecules. **Table 1** summarizes representative case studies from 2020 to 2024 in plant synthetic biology, including host species, engineering strategies, outcomes, and references. Strategic pathway engineering can achieve microgram-to-milligram yields per gram biomass with improved stability and yield. However, further optimization of metabolic flux and processes is necessary to achieve industrial-scale, cost-effective production.

**Table 1:** Representative applications of plant synthetic biology between 2020 and 2024.

Target compound	Host species (Chassis)	Engineering strategy	Outcome and scientific implication	Reference
Glucoraphanin	*Brassica* spp. *(N. benthamiana)*	Transient expression: The core glucoraphanin biosynthetic pathway (CGBP) 14 genes and *AtBCAT3, AtdCGS, AtIPMI2*, acyltransferase	4.74-fold increase compared to only CGBP genes expression (2.05 ± 0.32 µmol/g DW) → Identifies metabolic bottlenecks and auxiliary genes for enhanced production	Barnum et al. (2022)
Artemisinin	*C. morifolium* Ramat.	Artemisinin biosynthesis pathway multi-gene expression : *AaADS, AaCYP71AV1, AaCPR, AaDBR2, tHMGR*)	Accumulation of recombinant artemisinin in one of the transgenic chrysanthemum lines (W1, cv. White Snowdon) using GC-MS analysis → Demonstrates multi-gene engineering in ornamental host	Firsov et al. (2021)
Vinblastine precursors	*N. benthamiana*	Modular MIA pathway reconstitution : *CrSGD, CrGS, CrGO, CrRedox1,2, CrSAT*, etc.	Precondylocarpine acetate (PCA): 2.7 mg/g FT (Frozen Tissue); Catharanthine: 60 ng/g FT; Tabersonin: 10 ng/g FT → Enables early precursor accumulation in complex alkaloid pathway	Grzech et al. (2023)
Cannabinoid precursors	*N. benthamiana*	Transient expression : *CsAAE1, CsOLS, CsOAC, CsaPT4, CsTHCAS*	Olivetolic acid (OA) and OA-glucoside production → Expands the utility of plant chassis to cannabinoid biosynthesis	Gülck et al. (2020)
Anthocyanins	*Vaccinium myrtillus* *(N. benthamiana)*	Transcription factor + Transient overexpression (*VmMYBA1, VmMYBA2, VmMYBPA1.1 etc*)	Delphinidin 3-rutinoside: ≈ 20 AU/µg DW (*VmMYBA1)*, 6 AU/µg DW *(VmMYBA2*) Gallocatechin: ≈ 1.0 AU/µg DW (*VmMYBPA1.1+bHLH*) → Overexpression of specific MYB transcription factors increases anthocyanin and proanthocyanidin pathway metabolite levels	Karppinen et al. (2021)
Tanshinones/Phenolic acids	*Salvia miltiorrhiza*	Artificial Transcription Factor (*SmMYB36*-VP16) + hairy root system	Tanshinones: 6.44 mg/g DW; Phenolic acids: 141.03 mg/g DW → Enhances target compound yield via synthetic transcription factor	Jia et al. (2024)
Diosmin	*N. benthamiana*	Flavonoid multi-gene transient expression : *PAL, CHS, FNS, F3’H, OMT, etc.*	Diosmin: 61.9 nmol/g FW→ Achieves *de novo* biosynthesis of glycosylated flavone	Lee et al. (2024a)
Chrysoeriol	*N. benthamiana*	Flavonoid multi-gene transient expression : *AtPAL, OsCHS, OsFNS, OsF3’H, CrOMT2*	69.78 µg/g DW → Demonstrates methylated flavone biosynthesis in plant system	Lee et al. (2024b)
Saponin (QS-7)	*Quillaja saponaria* *(N. benthamiana)*	Transient expression of 19 genes from *Quillaja saponaria*: QA core pathway + glycosyltransferases + tailoring enzymes	QS-7 (QS saponin, Vaccine adjuvant): 7.9 µg/g DW → Enables scalable plant-based production of complex vaccine adjuvants	Reed et al. (2023)
Paclitaxel intermediate	*N. benthamiana*	Transient expression from *Taxus baccata* with *AtHMGR*, *AtBZO*, *Pc21g30650*	Baccatin III: 154.84 ng/g FW; 10-deacetylbaccatin III: 15.40 ng/g FW; Paclitaxel (with substrate feeding): 29.39 ng/g (*TmAAE16*), 64.29 ng/g (*Pc21g30650*); Identified a new C4b-C20 epoxidase → Demonstrates feasibility of plant chassis for anticancer precursor synthesis	Zhang et al. (2023)

This table presents representative applications of plant synthetic biology from the past five years (2020–2024), categorized by both the engineering strategies employed and the plant chassis used. The classification highlights how molecular tools such as transcription factor overexpression, multi-gene pathway reconstitution, and transient expression have been adapted across various host systems to optimize the biosynthesis of high-value compounds.

## Discussion

Plant synthetic biology has made significant progress; however, its widespread application remains limited by various technical, computational, and regulatory challenges. For instance, modular pathway reconstruction has been enabled by the DBTL framework. Nevertheless, the lack of real-time metabolic feedback control often results in suboptimal flux and yield (Goshisht, 2024). Also, the complex and context-dependent nature of plant metabolic networks means that identical interventions can yield different outcomes across species, tissues, and environments (Barnum et al., 2022; Han and Miao, 2024; Reed et al., 2023). Large-scale engineering typically causes flux imbalances, accumulation of toxic intermediates, and unexpected yield reductions, even when pathway gene optimization is applied (Han and Miao, 2024). The fact that many advances in plant synthetic biology remain at the proof-of-concept stage underscores the urgent need for reliable, field-ready systems capable of consistent and stable metabolite production in agricultural settings. Many proof-of-concept advances fail to scale effectively in agricultural or industrial contexts, underscoring the importance of incorporating scale-up considerations early in the design phase (Han and Miao, 2024).

As noted earlier, transformation and gene delivery barriers remain significant constraints in plant synthetic biology, particularly in species with recalcitrant regeneration systems (Altpeter et al., 2016). Overcoming these challenges is essential for translating genome editing successes into scalable, plant-based bioproduction platforms. Target selection remains challenging because many plant genomes are either un-sequenced or lack high-quality annotations (Cheng et al., 2025). Currently, transformation-free delivery systems and tissue culture-independent technologies, such as viral vectors and nanocarrier-based DNA delivery, are being developed to address these challenges (Zhou et al., 2023). As discussed above, *N. benthamiana* enables efficient transient expression but faces protein stability and consistency issues at an industrial scale. Selecting a production chassis requires considering metabolite stability, pathway length, and crosstalk, as these factors can also influence regulatory approval (Barnum et al., 2022; Han and Miao, 2024).

Regulation poses a significant challenge for synthetic plant biology. Regulations concerning genetically modified or genome-edited plants vary widely and are often in flux across many countries, which can hinder or delay the development and commercialization of new products. Moreover, the unique features of synthetic biology such as novel traits or entirely new metabolic pathways may not align with traditional risk assessment frameworks. Inconsistencies in national regulations can impact trade and global competitiveness, while regulatory gaps create uncertainty for developers. Although the foundational knowledge of plant synthetic biology has grown quickly, significant technical and regulatory hurdles still prevent its full translation to commercial and global applications (Atimango et al., 2024). Therefore, progress depends not only on technological or biological advances, but also on proactive policy alignment and stakeholder engagement (Bandyopadhyay et al., 2020). Bridging technical feasibility, computational predictability, and regulatory readiness is essential to ensure a smooth transition from the laboratory to the market.

## Future opportunities

Given these interconnected challenges, plant synthetic biology is a vital tool for enhancing biomolecule production and advancing environmentally sustainable agricultural practices (Atimango et al., 2024; Bandyopadhyay et al., 2020). Integrating technical, computational, and policy solutions from the outset is essential, as persistent bottlenecks hinder large-scale deployment (Altpeter et al., 2016). Long-term progress in plant synthetic biology requires interdisciplinary collaboration. To address challenges in plant systems, engineers, computer scientists, and plant biologists must work together to develop innovative technologies, such as context-independent and chassis-agnostic genetic constructs (Altpeter et al., 2016). Design efforts are facilitated by standardized modular cloning techniques like Golden Gate and Loop assembly. Additionally, AI-driven models are beginning to emerge that can predict pathway bottlenecks prior to physical prototyping (Goshisht, 2024). Integrating predictive modeling and AI-driven simulations with early-stage regulatory planning can reduce trial and error during development and accelerate readiness for industrial applications.

Therefore, predictive computational modeling, international standards, stakeholder frameworks, and the development of reliable, commercially deployable bio-foundries should be prioritized in synthetic biology. This coordinated approach will enable plant synthetic biology to have a revolutionary and long-lasting impact on food, health, and the green economy in the future (Han and Miao, 2024). Through these collaborative efforts, plant systems can enhance agricultural productivity, promote environmental sustainability, and facilitate the development of biomaterials (Atimango et al., 2024; Bandyopadhyay et al., 2020). By addressing both scientific and socioeconomic challenges, plant synthetic biology offers sustainable solutions for biotechnology and agriculture. Progress in this field is expected to accelerate with continued research and collaboration.

## References

[B1] Al Aboud N. M. (2024). Unlocking the genetic potential: Strategies for enhancing secondary metabolite biosynthesis in plants. J. Saudi Soc Agric. Sci. 23, 542–554. doi: 10.1016/j.jssas.2024.06.004

[B2] Altpeter F. Springer N. M. Bartley L. E. Blechl A. E. Brutnell T. P. Citovsky V.. (2016). Advancing crop transformation in the era of genome editing. Plant Cell 28, 1510–1520. doi: 10.1105/tpc.16.00196, PMID: 27335450 PMC4981132

[B3] Alvisi N. van Noort K. Dwiani S. Geschiere N. Sukarta O. Varossieau K.. (2021). β-Hexosaminidases along the secretory pathway of *Nicotiana benthamiana* have distinct specificities toward engineered helminth N-glycans on recombinant glycoproteins. Front. Plant Sci. 12. doi: 10.3389/fpls.2021.638454, PMID: 33815445 PMC8010188

[B4] Andrianantoandro E. Basu S. Karig D. K. Weiss R. (2006). Synthetic biology: New engineering rules for an emerging discipline. Mol. Syst. Biol. 2, 2006.02028. doi: 10.1038/msb4100073, PMID: 16738572 PMC1681505

[B5] Atimango A. O. Wesana J. Kalule S. W. Verbeke W. De Steur H. (2024). Genome editing in food and agriculture: From regulations to consumer perspectives. Curr. Opin. Biotechnol. 87, 103127. doi: 10.1016/j.copbio.2024.103127, PMID: 38564970

[B6] Baltes N. J. Voytas D. F. (2015). Enabling plant synthetic biology through genome engineering. Trends Biotechnol. 33, 120–131. doi: 10.1016/j.tibtech.2014.11.008, PMID: 25496918

[B7] Bandyopadhyay A. Kancharla N. Javalkote V. S. Dasgupta S. Brutnell T. P. (2020). CRISPR-Cas12a (Cpf1): A versatile tool in the plant genome editing tool box for agricultural advancement. Front. Plant Sci. 11. doi: 10.3389/fpls.2020.584151, PMID: 33214794 PMC7668199

[B8] Barnum C. R. Endelman B. J. Ornelas I. J. Pignolet R. M. Shih P. M. (2022). Optimization of heterologous glucoraphanin production *in planta* . ACS Synth. Biol. 11, 1865–1873. doi: 10.1021/acssynbio.2c00030, PMID: 35438493

[B9] Beritza K. Watts E. C. van der Hoorn R. A. L. (2024). Improving transient protein expression in agroinfiltrated *Nicotiana benthamiana* . New Phytol. 243, 846–850. doi: 10.1111/nph.19894, PMID: 38849321 PMC11494410

[B10] Brunk E. Chang R. L. Xia J. Hefzi H. Yurkovich J. T. Kim D.. (2018). Characterizing posttranslational modifications in prokaryotic metabolism using a multiscale workflow. Proc. Natl. Acad. Sci. U.S.A. 115, 11096–11101. doi: 10.1073/pnas.1811971115, PMID: 30301795 PMC6205427

[B11] Chavez B. G. Srinivasan P. Glockzin K. Kim N. Montero Estrada O. Jirschitzka J.. (2022). Elucidation of tropane alkaloid biosynthesis in *Erythroxylum coca* using a microbial pathway discovery platform. Proc. Natl. Acad. Sci. U.S.A. 119, e2215372119. doi: 10.1073/pnas.2215372119, PMID: 36442128 PMC9894180

[B12] Cheng L. T. Wang Z. L. Zhu Q. H. Ye M. Ye C. Y. (2025). A long road ahead to reliable and complete medicinal plant genomes. Nat. Commun. 16, 2150. doi: 10.1038/s41467-025-57448-8, PMID: 40032878 PMC11876585

[B13] Dai X. Shen L. (2022). Advances and trends in omics technology development. Front. Med. (Lausanne) 9. doi: 10.3389/fmed.2022.911861, PMID: 35860739 PMC9289742

[B14] Dudley Q. M. Jo S. Guerrero D. A. S. Chhetry M. Smedley M. A. Harwood W. A.. (2022). Reconstitution of monoterpene indole alkaloid biosynthesis in genome engineered *Nicotiana benthamiana* . Commun. Biol. 5, 949. doi: 10.1038/s42003-022-03904-w, PMID: 36088516 PMC9464250

[B15] Firsov A. Pushin A. Motyleva S. Pigoleva S. Shaloiko L. Vainstein A.. (2021). Heterologous biosynthesis of artemisinin in *Chrysanthemum morifolium* Ramat. Separations 8, 75. doi: 10.3390/separations8060075

[B16] Gharat S. A. Tamhane V. A. Giri A. P. Aharoni A. (2025). Navigating the challenges of engineering composite specialized metabolite pathways in plants. Plant J. 121, e70100. doi: 10.1111/tpj.70100, PMID: 40089911 PMC11910955

[B17] Golubova D. Tansley C. Su H. Patron N. J. (2024). Engineering *Nicotiana benthamiana* as a platform for natural product biosynthesis. Curr. Opin. Plant Biol. 81, 102611. doi: 10.1016/j.pbi.2024.102611, PMID: 39098308

[B18] Goshisht M. K. (2024). Machine learning and deep learning in synthetic biology: Key architectures, applications, and challenges. ACS Omega 9, 9921–9945. doi: 10.1021/acsomega.3c05913, PMID: 38463314 PMC10918679

[B19] Grzech D. Hong B. Caputi L. Sonawane P. D. O’Connor S. E. (2023). Engineering the biosynthesis of late-stage vinblastine precursors precondylocarpine acetate, catharanthine, tabersonine in *Nicotiana benthamiana* . ACS Synth. Biol. 12, 27–34. doi: 10.1021/acssynbio.2c00434, PMID: 36516122 PMC9872167

[B20] Gülck T. Booth J. K. Carvalho Â. Khakimov B. Crocoll C. Motawia M. S.. (2020). Synthetic biology of cannabinoids and cannabinoid glucosides in *Nicotiana benthamiana* and *Saccharomyces cerevisiae* . J. Nat. Prod. 83, 2877–2893. doi: 10.1021/acs.jnatprod.0c00241, PMID: 33000946

[B21] Guo W. Sheng J. Feng X. (2017). Mini-review: *In vitro* metabolic engineering for biomanufacturing of high-value products. Comput. Struct. Biotechnol. J. 15, 161–167. doi: 10.1016/j.csbj.2017.01.006, PMID: 28179978 PMC5288458

[B22] Han T. Miao G. (2024). Strategies, achievements, and potential challenges of plant and microbial chassis in the biosynthesis of plant secondary metabolites. Molecules 29, 2106. doi: 10.3390/molecules29092106, PMID: 38731602 PMC11085123

[B23] Hassan M. M. Zhang Y. Yuan G. De K. Chen J. G. Muchero W.. (2021). Construct design for CRISPR/Cas-based genome editing in plants. Trends Plant Sci. 26, 1133–1152. doi: 10.1016/j.tplants.2021.06.015, PMID: 34340931

[B24] Hassani D. Taheri A. Fu X. Qin W. Hang L. Ma Y.. (2023). Elevation of artemisinin content by co-transformation of artemisinin biosynthetic pathway genes and trichome-specific transcription factors in *Artemisia annua* . Front. Plant Sci. 14. doi: 10.3389/fpls.2023.1118082, PMID: 36895880 PMC9988928

[B25] Jia E. Li H. He F. Xu X. Wei J. Shao G.. (2024). Metabolic engineering of artificially modified transcription factor *SmMYB36*-VP16 for high-level production of tanshinones and phenolic acids. Metab. Eng. 86, 29–40. doi: 10.1016/j.ymben.2024.08.004, PMID: 39181435

[B26] Jiang X. Zhang Z. Wu X. Li C. Sun X. Wu F.. (2024). Heterologous biosynthesis of betanin triggers metabolic reprogramming in tobacco. Metab. Eng. 86, 308–325. doi: 10.1016/j.ymben.2024.11.002, PMID: 39505140

[B27] Jores T. Tonnies J. Wrightsman T. Buckler E. S. Cuperus J. T. Fields S.. (2021). Synthetic promoter designs enabled by a comprehensive analysis of plant core promoters. Nat. Plants 7, 842–855. doi: 10.1038/s41477-021-00932-y, PMID: 34083762 PMC10246763

[B28] Karppinen K. Lafferty D. J. Albert N. W. Mikkola N. McGhie T. Allan A. C.. (2021). MYBA and MYBPA transcription factors co-regulate anthocyanin biosynthesis in blue-coloured berries. New Phytol. 232, 1350–1367. doi: 10.1111/nph.17669, PMID: 34351627

[B29] Kim G. B. Kim H. R. Lee S. Y. (2025). Comprehensive evaluation of the capacities of microbial cell factories. Nat. Commun. 16, 2869. doi: 10.1038/s41467-025-58227-1, PMID: 40128235 PMC11933384

[B30] Kumar A. Anju T. Kumar S. Chhapekar S. S. Sreedharan S. Singh S.. (2021). Integrating omics and gene editing tools for rapid improvement of traditional food plants for diversified and sustainable food security. Int. J. Mol. Sci. 22, 8093. doi: 10.3390/ijms22158093, PMID: 34360856 PMC8348985

[B31] Lee S. B. Lee S. E. Lee H. Kim J. S. Choi H. Lee S.. (2024b). Engineering *Nicotiana benthamiana* for chrysoeriol production using synthetic biology approaches. Front. Plant Sci. 15. doi: 10.3389/fpls.2024.1458916, PMID: 39741678 PMC11685227

[B32] Lee H. Park S. Lee S. B. Song J. Kim T. H. Kim B. G. (2024a). Tailored biosynthesis of diosmin through reconstitution of the flavonoid pathway in *Nicotiana benthamiana* . Front. Plant Sci. 15. doi: 10.3389/fpls.2024.1464877, PMID: 39494057 PMC11527692

[B33] Li J. Mutanda I. Wang K. Yang L. Wang J. Wang Y. (2019). Chloroplastic metabolic engineering coupled with isoprenoid pool enhancement for committed taxanes biosynthesis in *Nicotiana benthamiana* . Nat. Commun. 10, 4850. doi: 10.1038/s41467-019-12879-y, PMID: 31649252 PMC6813417

[B34] Lin J. Yin X. Zeng Y. Hong X. Zhang S. Cui B.. (2023). Progress and prospect: biosynthesis of plant natural products based on plant chassis. Biotechnol. Adv. 69, 108266. doi: 10.1016/j.bioteChadv.2023.108266, PMID: 37778531

[B35] Luo Y. Li B. Z. Liu D. Zhang L. Chen Y. Jia B.. (2015). Engineered biosynthesis of natural products in heterologous hosts. Chem. Soc Rev. 44, 5265–5290. doi: 10.1039/c5cs00025d, PMID: 25960127 PMC4510016

[B36] Ma Z. Ma L. Zhou J. (2023). Applications of CRISPR/Cas genome editing in economically important fruit crops: recent advances and future directions. Mol. Hortic. 3, 1. doi: 10.1186/s43897-023-00049-0, PMID: 37789479 PMC10515014

[B37] Martin V. J. J. Pitera D. J. Withers S. T. Newman J. D. Keasling J. D. (2003). Engineering a mevalonate pathway in *Escherichia coli* for production of terpenoids. Nat. Biotechnol. 21, 796–802. doi: 10.1038/nbt833, PMID: 12778056

[B38] Molina-Hidalgo F. J. Vazquez-Vilar M. D’Andrea L. Demurtas O. C. Fraser P. Giuliano G.. (2021). Engineering metabolism in *Nicotiana* species: A promising future. Trends Biotechnol. 39, 901–913. doi: 10.1016/j.tibtech.2020.11.012, PMID: 33341279

[B39] Nonaka S. Arai C. Takayama M. Matsukura C. Ezura H. (2017). Efficient increase of γ-aminobutyric acid (GABA) content in tomato fruits by targeted mutagenesis. Sci. Rep. 7, 7057. doi: 10.1038/s41598-017-06400-y, PMID: 28765632 PMC5539196

[B40] Park S. Mani V. Kim J. A. Lee S. I. Lee K. (2022). Combinatorial transient gene expression strategies to enhance terpenoid production in plants. Front. Plant Sci. 13. doi: 10.3389/fpls.2022.1034893, PMID: 36582649 PMC9793405

[B41] Reed J. Orme A. El-Demerdash A. Owen C. Martin L. B. B. Misra R. C.. (2023). Elucidation of the pathway for biosynthesis of saponin adjuvants from the soapbark tree. Science 379, 1252–1264. doi: 10.1126/science.adf3727, PMID: 36952412

[B42] Rizzo P. Chavez B. G. Leite Dias S. D’Auria J. C. (2023). Plant synthetic biology: from inspiration to augmentation. Curr. Opin. Biotechnol. 79, 102857. doi: 10.1016/j.copbio.2022.102857, PMID: 36502769

[B43] Sadre R. (2024). Plant synthetic biology for human health: Advances in producing medicines in heterologous expression systems. Curr. Opin. Biotechnol. 87, 103142. doi: 10.1016/j.copbio.2024.103142, PMID: 38735192

[B44] Shen S. Zhan C. Yang C. Fernie A. R. Luo J. (2023). Metabolomics-centered mining of plant metabolic diversity and function: Past decade and future perspectives. Mol. Plant 16, 43–63. doi: 10.1016/j.molp.2022.09.007, PMID: 36114669

[B45] Singh K. S. van der Hooft J. J. J. van Wees S. C. M. Medema M. H. (2022). Integrative omics approaches for biosynthetic pathway discovery in plants. Nat. Prod. Rep. 39, 1876–1896. doi: 10.1039/d2np00032f, PMID: 35997060 PMC9491492

[B46] Sukenik S. C. Karuppanan K. Li Q. Lebrilla C. B. Nandi S. McDonald K. A. (2018). Transient recombinant protein production in glycoengineered *Nicotiana benthamiana* cell suspension culture. Int. J. Mol. Sci. 19, 1205. doi: 10.3390/ijms19041205, PMID: 29659495 PMC5979281

[B47] Wang Y. Gao R. Gu T. Li X. Wang M. Wang A.. (2025). Metabolomics and transcriptomics reveal the role of the terpene biosynthetic pathway in the mechanism of insect resistance in *Solanum habrochaites* . J. Agric. Food Chem. 73, 6253–6269. doi: 10.1021/acs.jafc.4c10397, PMID: 39998954

[B48] Wu G. Yan Q. Jones J. A. Tang Y. J. Fong S. S. Koffas M. A. G. (2016). Metabolic burden: cornerstones in synthetic biology and metabolic engineering applications. Trends Biotechnol. 34, 652–664. doi: 10.1016/j.tibtech.2016.02.010, PMID: 26996613

[B49] Yang Y. Chaffin T. A. Ahkami A. H. Blumwald E. Stewart C. N. Jr. (2022). Plant synthetic biology innovations for biofuels and bioproducts. Trends Biotechnol. 40, 1454–1468. doi: 10.1016/j.tibtech.2022.09.007, PMID: 36241578

[B50] Yeshi K. Crayn D. Ritmejerytė E. Wangchuk P. (2022). Plant secondary metabolites produced in response to abiotic stresses has potential application in pharmaceutical product development. Molecules 27, 313. doi: 10.3390/molecules27010313, PMID: 35011546 PMC8746929

[B51] Zhang Y. Wiese L. Fang H. Alseekh S. Perez de Souza L. Scossa F.. (2023). Synthetic biology identifies the minimal gene set required for paclitaxel biosynthesis in a plant chassis. Mol. Plant 16, 1951–1961. doi: 10.1016/j.molp.2023.10.016, PMID: 37897038

[B52] Zhang D. Xu F. Wang F. Le L. Pu L. (2025). Synthetic biology and artificial intelligence in crop improvement. Plant Commun. 6, 101220. doi: 10.1016/j.xplc.2024.101220, PMID: 39668563 PMC11897457

[B53] Zhou H. Eun H. Lee S. Y. (2023). Systems metabolic engineering for the production of pharmaceutical natural products. Curr. Opin. Syst. Biol. 37, 100491. doi: 10.1016/j.coisb.2023.100491

[B54] Zhu X. Liu X. Liu T. Wang Y. Ahmed N. Li Z.. (2021). Synthetic biology of plant natural products: From pathway elucidation to engineered biosynthesis in plant cells. Plant Commun. 2, 100229. doi: 10.1016/j.xplc.2021.100229, PMID: 34746761 PMC8553972

